# Use of anterolateral thigh flap for reconstruction of traumatic bilateral hemipelvectomy after major pelvic trauma: a case report

**DOI:** 10.1186/s40792-020-01009-2

**Published:** 2020-10-01

**Authors:** Saleh Al-wageeh, Faisal Ahmed, Khalil Al-naggar, Mohammad Reza Askarpour, Ebrahim Al-shami

**Affiliations:** 1 Department of General Surgery, Ibb University of Medical Science, Ibb, Yemen; 2 Department of Urology, Urology Research Center, Al-Thora General Hospital, Ibb University of Medical Science, Alodine Street, Ibb, Yemen; 3 Department of Urology, Urology Research Center, Al-Thora General Hospital, Ibb University of Medical Science, Ibb, Yemen; 4 Department of Urology, Shiraz University of Medical Sciences, Shiraz, Iran; 5 Department of Urology, Urology Research Center, Ibb University of Medical Science, Al-Thora hospital, Ibb, Yemen

**Keywords:** Amputation, Hemipelvectomy, Myocutaneous flap, Reconstruction, Trauma

## Abstract

**Background:**

Major pelvic trauma (MPT) with traumatic hemipelvectomy (THP) is rare, but it is a catastrophic health problem caused by high-energy injury leading to separation of the lower extremity from the axial skeleton, which is associated with a high incidence of intra-abdominal and multi-systemic injuries. THP is generally performed as a lifesaving protocol to return the patient to an active life.

**Case report:**

A 12-year male patient exposed to major pelvic trauma with bilateral THP survived the trauma and multiple lifesaving operations. The anterolateral thigh flap is the method used for wound reconstruction. The follow-up was ended with colostomy and cystostomy with wheelchair mobilization. To the best of our knowledge, there have been a few bilateral THP reports, and our case is the second one to be successfully treated with an anterolateral thigh flap.

**Conclusion:**

MPT with THP is the primary cause of death among trauma patients. Life-threatening hemorrhage is the usual cause of death, which is a strong indication for THP to save life.

## Introduction

Major pelvic trauma (MPT) associated with traumatic hemipelvectomy (THP) was described first by Turnbull in 1978 [1]. Although rare, it is a catastrophic health problem caused by high-energy injury leading to separation of the lower extremity from the axial skeleton from two joints [the symphysis pubis and the sacroiliac (SI) joint]. It is either incomplete (when a soft tissue still attaches the limb) or complete when the limb is separated without any soft-tissue attachment. These injuries are considered massive pelvic injuries [2].

A few victims survive these injuries, and the actual incidence is unknown, but it is usually underestimated [3]. Massive bleeding (approximately 3–4 L) can occur before the venous tamponade's effect, especially if there is significant pubic symphysis diastasis. Complex pelvic fractures are associated with a high incidence of intra-abdominal injuries (30%) and multisystem trauma (80%), determining the outcome of these injuries [4]. The primary associated intra-abdominal injuries are bladder and urethral injuries, and less common injuries include injuries to the liver, small bowel, spleen, and diaphragm [4]. Initial goals of management include control of life-threatening hemorrhage and patient stabilization followed by thorough debridement of the wound. All devitalized soft tissues must be excised sharply [2]. Conversely, viable muscle and fasciocutaneous tissue should be maintained for possible use in the definitive reconstruction surgery. THP is generally performed as a lifesaving protocol to return the patient to the active life, but when limb loss is inevitable, immediate amputation is better than the watchful waiting approach [4].

To the best of our knowledge, there are only few cases reported about bilateral THP. Here, we report a case of MPT associated with THP treated with an anterolateral thigh flap with success until full recovery.

## Case report

**Fig. 1:**
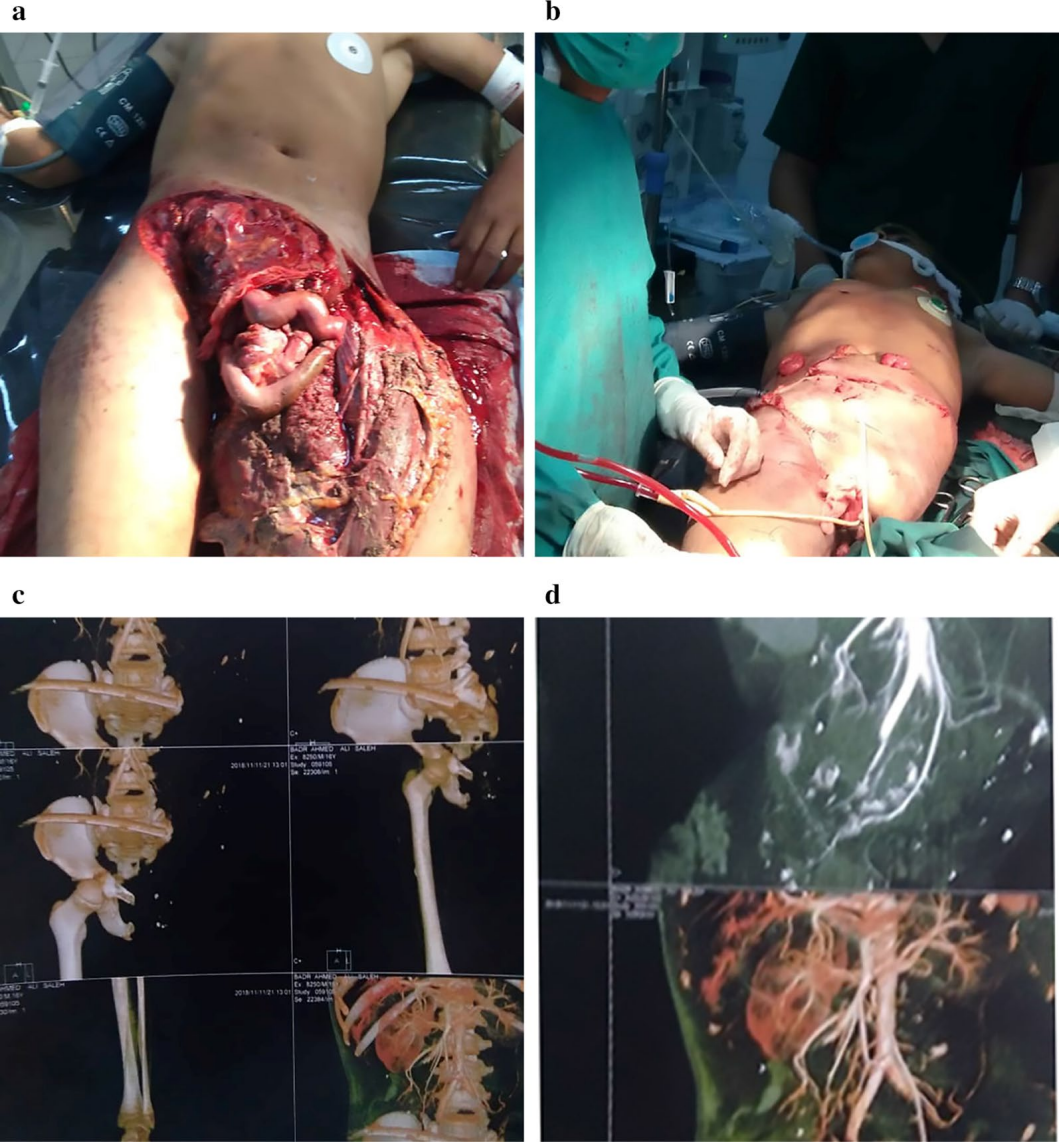
**a** Details of the wound on the left pelvic side before the initial operation [lower abdomen with exposed urinary bladder, eviscerated bowel, destructed perineum, and almost complete avulsion of the left lower extremity]. **b** Postoperative clinical situation with left traumatic hemipelvectomy and an ipsilateral anterolateral thigh flap, achieving the primary closure of the wound. **c** Radiological study showing dislocation of the right sacroiliac joint and symphysis pubis with only some ligamentous attachment. **d** Computed tomography angiography of the pelvis and right lower extremity indicating the absence of blood flow to the right lower extremity

A previously healthy 12-year-old boy was involved in a motorcycle accident. He was an unrestrained rear-seat passenger ejected from the motorcycle to meet a large vehicle passed over his lower abdomen and pelvis. The patient was admitted to the emergency department after 2 h of the accident. He was semi-conscious, slightly oriented, and pale, with a patent airway and the Glasgow Coma Scale score 13. His blood pressure was 90/50 mmHg with a pulse of 120 beats per minute, a breathing rate of 24 respirations per minute, and an oxygen saturation level of 94%. Resuscitation was started immediately with large pore peripheral two intravenous cannulas, and crystalloid solutions (1000 cc of R/L) were started, and blood was sent for routine investigations and cross-matching for blood transfusion. Examination revealed a degloved lower abdomen with exposed urinary bladder, eviscerated bowel, destructed perineum (including anus, rectum, and external genitalia: no palpable pulse, no motor function or sensation in both lower limbs and no active bleeding from the wound. The left lower limb was already disarticulated from the pelvis and only attached by soft tissues, which was considerably destroyed (Fig. 1a).

**Fig. 2:**
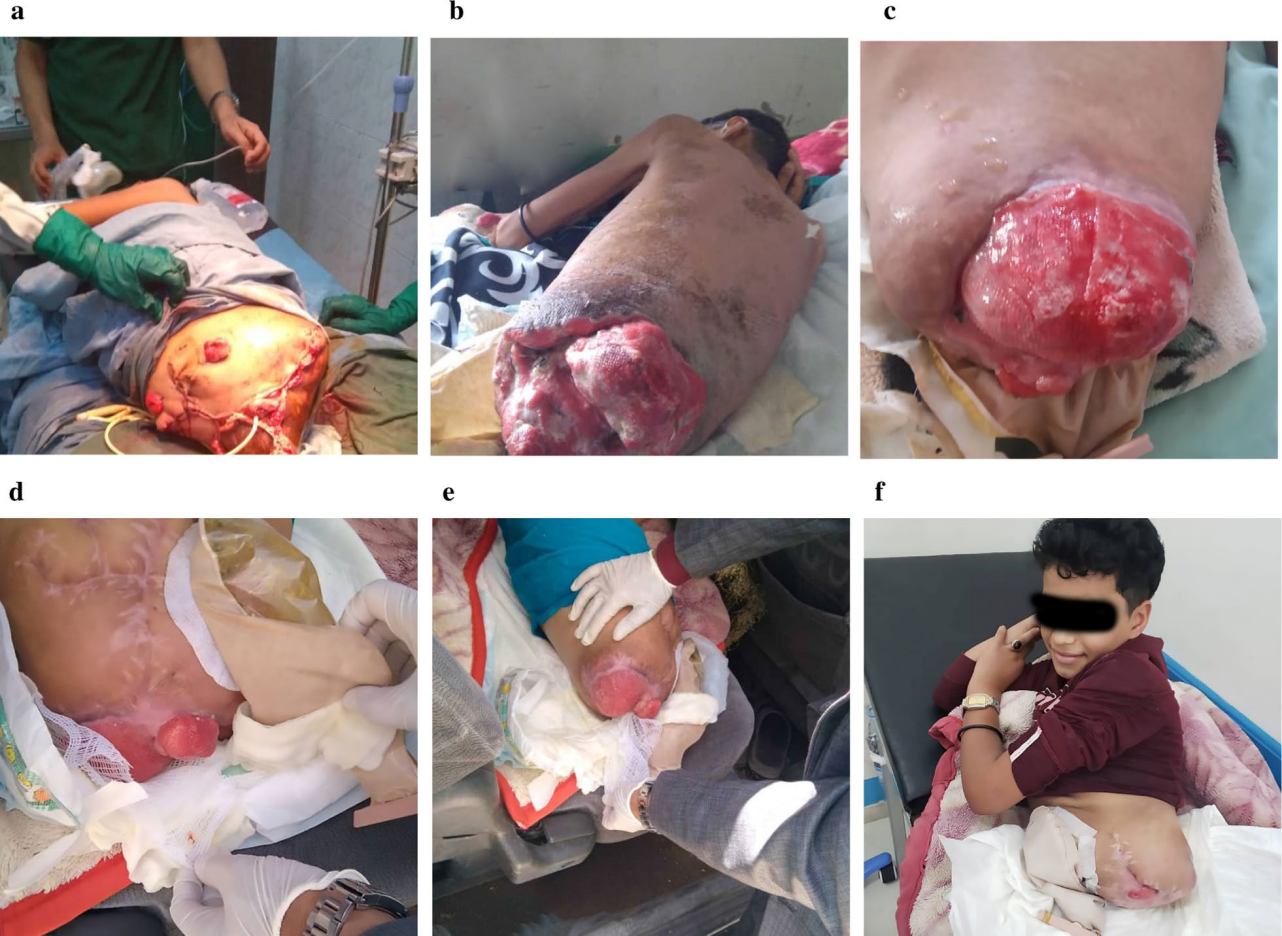
**a** Postoperative clinical situation achieving primary closure of the wound with completed amputation of both lower extremities. **b** and **c** Clinical photographs showing an anterolateral thigh flap with progressive skin necrosis. **d** Clinical follow-up 5 months after debridement of the remaining a gluteus myocutaneous flap; in the supine position, epithelialization was achieved. **e** In the lateral position, epithelialization was achieved. **f** Rehabilitation was started, along with the use of wheelchair prosthesis

The multidisciplinary team was consulted, including a general surgeon, orthopedist, urologist, and vascular surgeon. Blood investigations showed Hb:10 g/dl, WBC: 14,000 cell/μl, blood sugar: 90 mg/l, urea: 40 mg/dl, and creatinine: 0.9 mg/dl. He prepared for surgical exploration. Under general anesthesia, exploration reveals incomplete open separation of the left limb from the sacroiliac joint and symphysis pubis, complete thrombosis of external and internal iliac vessels, and major soft-tissue destruction, including transection of sciatic and femoral nerves. Amputation was completed (Fig. 1b). Exploration of the right side of the pelvis revealed thrombosis of the external iliac artery, intact internal iliac artery, and unstable right hemipelvis with a threatened limb, which was temporarily revascularized. Visceral injuries include the urinary bladder with complete urethral avulsion from the bladder neck, which are treated with debridement and repair carried out with suprapubic and bladder neck catheterization. Exploration of the wound and opening of the abdomen with a reduction of eviscerated bowel revealed that approximately 120 cm of the distal small bowel was injured with avulsed mesentery; therefore, resection was performed with end ileostomy and loop colostomy from the sigmoid colon. Perineal injury included complete amputation of the penis; the testicles, anus, and rectum were completely destructed. Extensive debridement, copious irrigation of the wound, and damage control closure with the utilization of an anterolateral myocutaneous thigh flap were carried out. By the end of the procedure, the patient received four units of packed cell, four units of fresh frozen plasma, four units of platelets, 2000 cc of crystalloid normal silane solution, and intravenous antibiotics (1-g cefepime and 500 mg metronidazole). The patient was transferred to the intensive care unit (ICU) to maintain his health status. After 12 h, the patient was stabilized, and computed tomography angiography of the pelvis was performed and it revealed disruption of the right sacroiliac joint and symphysis pubis with only some ligamentous attachment in addition to the absence of blood flow to the right lower extremity (Fig. 1c and d). After 24 h, the limb becomes cold and mottled appearance. Therefore, the right-side hemipelvectomy was carried out with an anterolateral myocutaneous thigh flap reconstruction. Two units of the packed cell were given to him during operation (Fig. 2a).

Forty-eight hours later, the flap started to become necrotic with some demarcation (Fig. 2b); as a result, the patient was prepared for debridement under general anesthesia (Fig. 2c). The patient was observed in the surgical ICU for 1 week, with two times debridement of the wound and daily flap irrigation. The patient was transferred to the general surgery ward with continuation of dressing every day. Three weeks later, the patient became malnourished owing to high output fistula, so that the patient was prepared for elective ileostomy closure, and ileocolic anastomosis was performed. Additionally, the end of colostomy was reconstructed from the previous loop sigmoid colostomy. Finally, the patient had end sigmoid colostomy, bladder neck cystostomy (bladder pouch), and wheelchair mobilization.

The patient recovered well, then underwent serial dressing in the surgical ward and discharged on day 45 with regular dressing performed in an outpatient clinic. On day 128, the wound became fully granulating (Fig. 2d and e), and the patient was admitted to a specialized center for skin grafting (Fig. 2f).

## Discussion

MPT associated with THP or pelvic fractures is the primary cause of death among trauma patients [4]. It is caused by high-energy trauma and associated with injury to other parts and systems of the body. The mortality after MPT associated with THP is estimated to be between 60 and 100% [4]. The actual incidence of survival is unknown. Associated injuries, which are common, especially anorectal (60%), genitourinary (85%), and adjacent bony structures, can affect the outcome of these traumas [5]. Early deaths are attributed to hemorrhage or central nervous system injuries, while delayed deaths are due to sepsis and multiple organ failure [3].

Spontaneous thrombosis of iliac vessels, extreme vasoconstriction, and vessel retraction into the pelvis causes survival after THP [6]. In our unusual case, the left external and internal iliac arteries were thrombosed, in addition to the right external artery, which was also thrombosed without vital organ injuries that may have saved our patient's life.

Pelvic visceral injuries are well documented with THPs, and the most common associated intra-abdominal injuries after pelvic viscera are injuries to the liver, small bowel, spleen, and diaphragm; these depend on the nature and severity of injury [3].

The priority in the management of such injuries is to save the patient's life. As described in the literature, amputation or limb salvage is a difficult decision, since no validated scoring system contributes to decision-making [7]. However, there are some apparent indications for amputation to save a life over limb, including life-threatening hemorrhage, gross wound contamination, and complete arterial, venous, and neurological injuries. Nonetheless, we amputated both limbs to save our patient's life. However, it was delayed on the right side for possible fixation, but vascular injury and limb ischemia with failure of temporary revascularization hindered us, and complete amputation was chosen. We delayed the decision on the right side, since it was a closed type without obvious soft-tissue injury and no bleeding, and the vascular injury was encompassed by temporary revascularization. This delay facilitates more investigations, decreases the operation time, and causes massive transfusion. There is a case of replantation with success following THP [8], but it results in paralytic limb and loss of sensation. Contrary to what is usually done and mentioned on many pieces of literature, diverting stoma in this case was carried out in the first operation, since the small bowel was eviscerated, and mesentery was avulsed, so that if delayed, it would have caused bowel ischemia and more sepsis [4].

Phantom limb pain, flap failure, sepsis, and vesicocutaneous or urethrocutaneous fistulas are the most common complications after MPT and THP [9]. Our patient developed flap necrosis, which was treated with repeated debridement resulting in a large raw area covered with skin graft after full granulation. Shrinkage of urinary bladder size due to a long time of exposure and vesicocutaneous fistula towing to complete loss of the urethra from the bladder neck are urinary tract complications, which will be managed by ureteric diversion later on life as the patient and relatives wish.

To the best of our knowledge, there were only few cases reported with successful reconstruction with an anterolateral myocutaneous flap [3]. In our case, we preserved the viable soft tissue as much as possible to cover the defect, and the loss of pelvic bone facilitated closure. Unfortunately, the weight-bearing and maintaining balance with a prosthesis will be decreased [3].

Other different reconstructive procedures were described in the literature. However, unfortunately, patients with THP usually have massive tissue destruction, and the use of a thoracoabdominal flap is avoided in patients with bilateral THP, since those patients need a strong musculature to hold their body and use a wheelchair.

D’Alleyrand et al. mentioned the principal management in patients with a THP, stressing on meticulous wound debridement, robust soft-tissue coverage, and excellent patient care through a multidisciplinary approach [10]. Malnutrition does not usually occur in pelvic trauma except if it is associated with high output fistula due to a complicated intra-abdominal process or massive bowel loss as in our patient who lost approximately 120 cm of distal small bowel, leading to proximal stoma formation.

As mentioned in many pieces of literature, after a full recovery, the patient stays in a rehabilitation unit, which is a usual way to train a disabled patient for his physical activities; we discharged our patient, since we had no rehabilitation unit. However, he developed excellent interaction with his wheelchair prosthesis and did his daily activities well [3].

## Conclusion

MPT with THP is the primary cause of death among trauma patients. Life-threatening hemorrhage is the usual cause of death, which is a strong indication for THP to save a life. Bilateral THP is unusual, as in our case, which was treated with a multidisciplinary team ended by colostomy and cystostomy with wheelchair mobilization. We described a challenging case of a 12-year-old boy successful reconstruction of the bilateral pelvis using an anterolateral thigh flap.

## Data Availability

All related data are included within the article.

## Abbreviations

ICU: Intensive care unit
MPT: Major pelvic trauma
SI: Sacroiliac
THP: Traumatic hemipelvectomy
